# The prevalence of overactive bladder and its impact on the quality of life: A cross-sectional study

**DOI:** 10.1080/2090598X.2023.2221403

**Published:** 2023-06-10

**Authors:** Shrouq Qudah, Mohammad Abufaraj, Randa Farah, Abdulrahman Almazeedi, Ali Ababneh, Mazen Alnabulsi, Ayman Qatawneh, Dana Hyassat, Kamel Ajlouni

**Affiliations:** a School of Medicine, The University of Jordan, Amman, Jordan; b Division of Urology, Department of Special Surgery, Jordan University Hospital, Amman, Jordan; c Department of Internal Medicine, School of Medicine, Jordan University Hospital, Amman, Jordan; d Department of Obstetrics and Gynecology, Faculty of Medicine, The University of Jordan, Amman, Jordan; e The National Center for Diabetes, Endocrinology and Genetic (NCDEG)/The University of Jordan, Amman, Jordan

**Keywords:** Overactive Bladder, quality of life, urge incontinence

## Abstract

**Objective:**

Overactive bladder (OAB) is a common condition affecting both men and women and has been shown to affect the quality of life. We conducted this study to estimate the prevalence of OAB, and to incorporate symptom severity, symptom bother and health-related quality of life (HRQL) in the assessment of OAB and evaluate associated factors.

**Methodology:**

A total of 940 participants were categorized into non-OAB and OAB using the Overactive Bladder Symptom Score (OABSS). HRQL and symptom bother were measured using the Overactive Bladder Questionnaire – Short Form (OAB-q SF). Descriptive analyses and multivariable regression analyses were performed.

**Results:**

The prevalence of OAB among our population was 27.4%. Patients with older age (Odd ratio [OR] = 2.26, 95% confidence interval [CI]: 1.6–3), higher body mass index (BMI) (OR = 2.6, 95% CI: 1.8–3.8), comorbidities (OR = 2.6, 95% CI: 1.9–3.5) and history of recurrent urinary tract infection (UTI) s (OR = 1.9, 95% CI: 1.4–2.6) were significantly associated with increased risk of OAB (*p* < 0.001). The mean OAB symptom bothers score was 35.7 + 22.9 and increased significantly across OAB severity groups (*p* < 0.001). The mean HRQL score was 73.3 + 22 and a significant decreased across OAB severity groups (*p* < 0.001). All OAB symptoms showed significant positive correlation with increased symptom bother (*p* < 0.001) in addition to significant inverse correlation with HRQL (*p* < 0.001).

**Conclusion:**

OAB is a prevalent condition in our population and the associated symptoms negatively affect HRQL. In this study, the detrimental effect is not exclusive to UUI and can be attributed to the other elements in the symptom spectrum of OAB. Screening for OAB should be considered during routine clinical visits using validated and reliable measures for early detection of symptoms and possible modification of risk factors to improve the outcome.

## Introduction

Overactive bladder (OAB), as defined by the international continence Society (ICS), is a complex of symptoms characterized by the presence of urgency with or without urinary incontinence (UI), and often with frequency and nocturia, provided the absence of underlying infections or pathologies [1]. OAB is an extremely common urological disease affecting both men and women [2]. OAB adversely affects the quality of life, throughout the physical, mental, emotional and sexual domains [2–5]. While UI has been the forefront in research in evaluating health-related quality of life (HRQL), other investigators demonstrated that bother and HRQL can be significantly influenced by urgency [3,6], even more than UI. The symptomatic definition of OAB helps in establishing an initial diagnosis which has allowed for early management [7]. Yet, rates in seeking healthcare remains low [8]. Studies investigating treatment seeking behavior have shown that individuals affected with OAB misinterpret their urinary symptoms as a normal part of aging [9] or lack awareness about the availability of treatment [10]. Coping strategies including fluid restriction, physiotherapy and the use of absorbent pads to adapt with the urinary symptoms have also been shown to interfere with daily and social activities [9,11]. OAB forms a significant financial burden and was estimated to contribute to a direct cost of 1.2–2.7 trillion in 2008 [12]. In the United States, the health care costs were 2.5 times higher in OAB patients compared to their counterparts [13]. In addition to health care, OAB has been estimated to have an indirect economical cost of 841 million dollars by adversely impacting work productivity [14]. Despite the highest predicted burden of OAB to be in Asia [12], data surrounding OAB remains scarce in Jordan. The focus has mainly been directed towards UI [15], or OAB among women aged ≥40 [16]. Consequently, we aim to assess the prevalence of OAB among men and women aged ≥18, provide an insight on the extent of symptom bother, as well as integrate quality of life as an outcome measure in the assessment of OAB. Secondarily, we aim to evaluate associated factors, and explore trends in treatment seeking behaviors among affected individuals.

## Materials and methods

### Patients and settings

This was a cross-sectional study performed at the outpatient clinics of Jordan University Hospital (JUH); a tertiary hospital and The National Center for Diabetes, Endocrinology and Genetic (NCDEG); a healthcare center, both located in Amman, the capital city of Jordan. Data was collected between October 2021 and January 2022. The necessary sample size was calculated by projecting an estimated prevalence of 25%, to achieve a 95% CI, and a margin of error of 5%. Assuming a response rate of 60%, we concluded that our sample should constitute 860 individuals. Conservatively, data collection was continued until 1000 participants were reached.

#### Inclusion and exclusion criteria

Inclusion criteria instated the participants to be ≥18 years old, capable of understanding the questionnaire and providing consent. Generally, all those who had any neurological disorder (i.e. Multiple Sclerosis/Parkinson’s/spinal cord injury/stroke) and who reported a recent urinary tract infection (UTI) (current UTI or UTI within the past month) whether clinically diagnosed or self-reported as ‘burning or painful sensation upon urination’, current renal stones, recent urological surgery (within the last six months), history of urological cancer and radiotherapy were considered ineligible to proceed with the survey analysis . Additionally, female participants with pelvic organ prolapse (either self-reported or clinically diagnosed), current pregnancy, history of gynecological cancer and male participants who had benign prostatic hyperplasia (BPH) diagnosed clinically or receiving medications were excluded from the study.

#### Measurements

Participants were first introduced to the purpose of the study and the nature of the questionnaire. Following this, a written consent was obtained to proceed with the completion of the survey. The survey was self-administered with a trained healthcare personnel present to provide any non-influential assistance, if requested. Study participants filled out a data sheet regarding socioeconomic and demographic information (age, sex, marital status, household income), as well as health-related and lifestyle characteristics which included questions about the presence of any comorbidities such as: diabetes, hypertension, anxiety or depression, and history of recurrent UTIs. Weight and height (for body mass index (BMI) calculation), smoking status, fluid, and caffeine intake were also obtained. The Overactive Bladder Symptom Score (OABSS) was the diagnostic tool of choice in our study. The OABSS was developed and validated to quantify OAB symptoms, as defined by the ICS, using four questions asking about the occurrence frequency of: urinary urgency, daytime frequency, nocturia, and urge urinary incontinence (UUI) over the past week. Each reported answer is designated a score, and the resulting sum score can range from 0 to a maximum score of 15; higher scores on the OABSS were also indicative of higher OAB symptom severity [17]. In 2019, a reliable and valid Arabic version of the OABSS was produced [18], allowing for the assessment of OAB symptoms in Arabic-speaking populations. Accordingly, we utilized the Arabic version and categorized our study participants into two groups (OAB and non-OAB) depending on their OABSS sum score. OAB was defined as a total score ≥ 3, and a score on the urgency question ≥ 2 (urgency occurring once a week or more). This was in accordance with the ICS that integrated urgency as a key characteristic in the OAB symptomatic definition [1]. Additionally, the OAB group was subcategorized into mild OAB (total score ≤ 5), moderate (6–11), and severe (≥12). Any individual who met the criteria for OAB and scored ≥ 1 on the urge UI questions was considered to have OAB with UI. In our study, OAB without UI is referred to as OABdry and OAB with UI is referred to as OABwet. For the evaluation of HRQL and symptom bother, we utilized the Overactive Bladder Questionnaire – Short Form (OAB-q SF). The OABq – SF is a valid and reliable condition-specific instrument that consists of 19 items divided into two parts; six items asking about OAB symptom bother and 13 items asking about OAB impact on different HRQL domains (sleep, social, concern and coping) over the past month [19]. All 19 items were designated to a Likert scale ranging from a score of 1 to 6. The results of the questionnaire were interpreted and transformed from crude scores into a value out of 100. For symptom bother, higher scores were suggestive of increased symptom bother as opposed to the HRQL where higher scores were suggestive of a better outcome. The questionnaire was translated to the Arabic language, prior to administration, according to the Good Practice Guidelines [20], where linguistic and cultural integrity were ensured. Healthcare seeking behavior was explored by asking participants if they had ever sought medical advice regarding their urinary symptoms, had been previously diagnosed with OAB or if they had received treatment. Respondents who had received treatment were asked to specify the type and state of treatment (i.e. behavioral, pharmacotherapy/current, previous).

#### Statistical analysis

All analyses were performed using Stata version 14. Data were first inserted into an Excel spreadsheet, and the exclusion criteria, mentioned above, was implemented. Descriptive statistics using frequencies and percentages were used to express categorical variables. Continuous data were expressed as means ± SD and medians with IQR following the normalcy of their distribution. Chi square tests and Mann-Whitney U test were performed to determine the association with OAB. Logistic regression was performed and odds ratio with 95% CI were produced. Following the unadjusted regression, all factors that reached statistical significance (p < 0.05) were fit into the final logistic model. Multivariable linear regression analysis was performed for symptom correlation with bother and HRQL. *P* value < 0.05 was considered significant. Regarding reliability of study instruments, Cronbach alpha ≥ 0.70 was used to define good internal consistency.

#### Ethical considerations

The study was approved by the Institutional Review Board (IRB) of the Faculty of Medicine and Jordan University Hospital at The University of Jordan (309/2021) and the IRB at the National Center for Diabetes and Endocrinology and Genetics (NCDE) . Participants were assured confidentiality of information.

## Results

### Reliability of measure*s*

The OABq – SF showed good reliability and internal consistency; Cronbach alpha for six items measuring the degree of bother was 0.88, for 13 items measuring the HRQL was 0.95 and for the entire questionnaire (19 items) was 0.96. For the OABSS, Cronbach alpha was 0.77.

### Baseline characteristics

A total of 1,125 participants were recruited. Following the exclusion criteria, 185 participants were found ineligible for further analysis providing a response rate of 83.5%. The final number of eligible study participants was 940. Table 1 shows the baseline characteristics and association with OAB. Overall, the median age of the respondents was 43 years, female to male ratio was 1.1, 62.5% were married, 54.2% were non-workers, housewives or retired and the household income was > 500 (JDs) among 512 individuals (54.4%). The majority (77.5%) had received an education level equivalent to college or higher. Smoking was reported in 28.6% of study individuals while caffeine intake (i.e. coffee, tea) was reported in 89.8%. More than half of the study participants (66.9%) had an average fluid intake of less than 2500 (mL). The presence of comorbidities, previous history of renal stones and previous history of UTIs were observed in 52.6%, 9%, and 36.1% of the study population, respectively.

**Table 1.** Baseline characteristics of 940 study participants.

Baseline characteristics		Overall n = 940%)	Non-OAB n = 682 (72.6%)	OAB n = 258 (27.4%)	*p* value
Age, median (IQR)		43 (30)	39 (30)	50.5 (22)	<0.001*
Age group+	**<****40**	436 (46.4)	362 (83)	74 (17)	<0.001*
	**>40**	504 (53.6)	320 (63.5)	184 (35.5)	
Gender	Male	446 (47.4)	348 (78)	98 (22)	<0.001*
	Female	494 (52.6)	334 (67.6)	160 (32.4)	
Marital status	Married	588 (62.5)	399 (67.9)	189 (32.1)	<0.001*
	Single/divorced/widow	352 (37.4)	283 (80.4)	69 (19.6)	
Education	Elementary school	38 (4)	26 (68.4)	12 (31.6)	0.8
	Secondary school	173 (18.4)	126 (72.8)	47 (27.2)	
	College and higher	729 (77.5)	530 (72.7)	199 (27.3)	
Employment	Currently employed	449 (47.8)	304 (67.7)	145 (32.3)	0.01*
	Retired/housewives/unemployed	379 (40.3)	287 (75.7)	92 (24.3)	
Income+	<500 JD	428 (45.5)	296 (69.2)	132 (30.8)	0.03*
	≥500 JD	512 (54.5)	386 (75.4)	126 (24.6)	
BMI+	<25	289 (30.7)	238 (82.4)	51 (17.6)	<0.001*
	25–29	335 (35.6)	243 (72.5)	92 (27.5)	
	≥30	311 (33)	198 (63.7)	113 (36.3)	
Smoking (Yes)		269 (28.6)	200 (74.4)	69 (25.6)	0.4
Fluid intake+	<2500	629 (66.9)	468 (74.4)	161 (25.6)	0.07
	>2500	311 (33)	214 (68.8)	97 (31.2)	
Caffeine intake (Yes)		845 (89.9)	605 (71.4)	240 (28.4)	0.05
Medical Comorbidities (Yes)		495 (52.6)	316 (63.8)	179 (36.2)	<0.001*
Previous UTI		340 (36)	217 (63.8)	123(36.2)	<0.001*
Previous renal stones		85 (9)	55 (64.7)	30 (35.3)	0.09

### Prevalence of OAB

We found that the overall prevalence of OAB was 27.4% (258), 22% among men and 32.4% among women. Almost half of the OAB patients (51.2%) had OAB_wet_ with the highest prevalence (28%) among the eldest age group (≥70 years) and lowest (4.5%) in the youngest age group (18–29).

In women with OAB, 56.8% had urge UI compared to 41.8% of men with OAB (*p* = 0.01). Mild OAB was observed in 36.4% of affected individuals, moderate OAB in 12.7% while 50.7% had severe OAB.

### Factors associated with OAB

Results of the regression model are shown in Table 2. We performed bivariate regression analysis and found that age (Odd ratio [OR] = 2.26, 95% confidence interval [CI]: 1.6–3), higher BMI (OR = 2.6, 95% CI: 1.8–3.8), presence of comorbidities (OR = 2.6, 95% CI: 1.9–3.5) and history of recurrent UTIs (OR = 1.9, 95% CI: 1.4–2.6) were all significantly associated with increased risk of OAB (*p* < 0.001 for all). Additionally, men as compared to women, and those who were single, divorced or widowed as compared to those who were currently married had two times decreased odds of OAB (*p* < 0.001 for both). Employment (OR = 0.6, 95% CI: 0.4–0.9) and income > 500 (OR = 0.7, 95% CI: 0.5–0.9) were significantly associated with decreased odds of OAB (*p* = 0.01, *p* = 0.03, respectively). On the other hand, educational level, smoking, caffeine intake as well as fluid intake did not reach statistical significance. For the multivariable regression model, gender, BMI, presence of comorbidities, and history of recurrent UTIs remained significantly associated with increased risk of OAB (*p* < 0.05). Considering the pertinent effect of fluid intake, it was included in the final regression model despite not yielding prior statistical significance, this showed that fluid intake > 2500 (mL) might increase the odds of OAB (OR = 1.39, 95% CI: 0.9–1.9) with *p*-value of 0.05, yet not statistically significant in our analysis.

**Table 2.** Logistic regression analysis for factors predictive of OAB.

Variable	OR (95% CI)	*P* value	AOR (95% CI)	P value
**Age (ref. <45)**^+^				
≥45	2.26 (1.6–3)	<0.001*	1.13 (0.7–1.7)	0.5
**Gender (ref. female)**				
Male	0.5 (0.4–0.7)	<0.001*	0.5 (0.3–0.7)	0.001*
**Marital status (ref. Married)**				
Single/divorced/widowed	0.5 (0.3–0.7)	<0.001*	1.3 (0.8–1.9)	0.1
**Educational level (ref. Elementary)**				
Secondary	0.7 (0.3–1.7)	0.5		
College/University or higher	0.7 (0.3–1.5)	0.4		
**Income (ref. <500)**^+^				
>500	0.7 (0.5–0.9)	0.03*	0.7 (0.5–1)	0.05
**Occupation (ref. Unemployed/retired/housewife)**				
Employed	0.6 (0.4–0.9)	0.01*	1.2 (0.8–1.7)	0.3
**BMI (ref. <25)**^+^				
25 - <30	1.7 (1.2–2.5)	0.01*	1.6 (1.03–2.56)	0.05
≥30	2.6 (1.8–3.8)	<0.001*	1.8 (1.1–2.9)	0.01*
**Fluid intake (ref. <2500)**^+^				
≥2500	1.3 (0.9–1.7)	0.07	1.39 (0.9–1.9)	0.05
**Caffeine intake (ref. none)**				
Yes	1.6 (0.9–2.8)	0.05		
**Smoking (ref. non-smoker/Ex-smoker)**				
Smoker	0.8 (0.6–1.2)	0.4		
**Comorbidities (ref. none)**				
Yes	2.6 (1.9–3.5)	<0.001*	1.86 (1.2–2.8)	0.01*
**History of recurrent UTIs (ref. none)**				
Yes	1.9 (1.4–2.6)	<0.001*	1.6 (1.1–2.2)	0.01*

*reference, OAB*: Overactive bladder *BMI: body mass index, UTIs: Urinary Tract Infections*.

^*+*^*Age expressed in years; BMI expressed in Kg/M*^*2*^; *Income expressed in Jordanian Dinars (JDs); fluid intake measured in mL*.

*Statistically significant P value < 0.05.

### OAB symptoms, severity, degree of bother and HRQL

**Figure 1.**
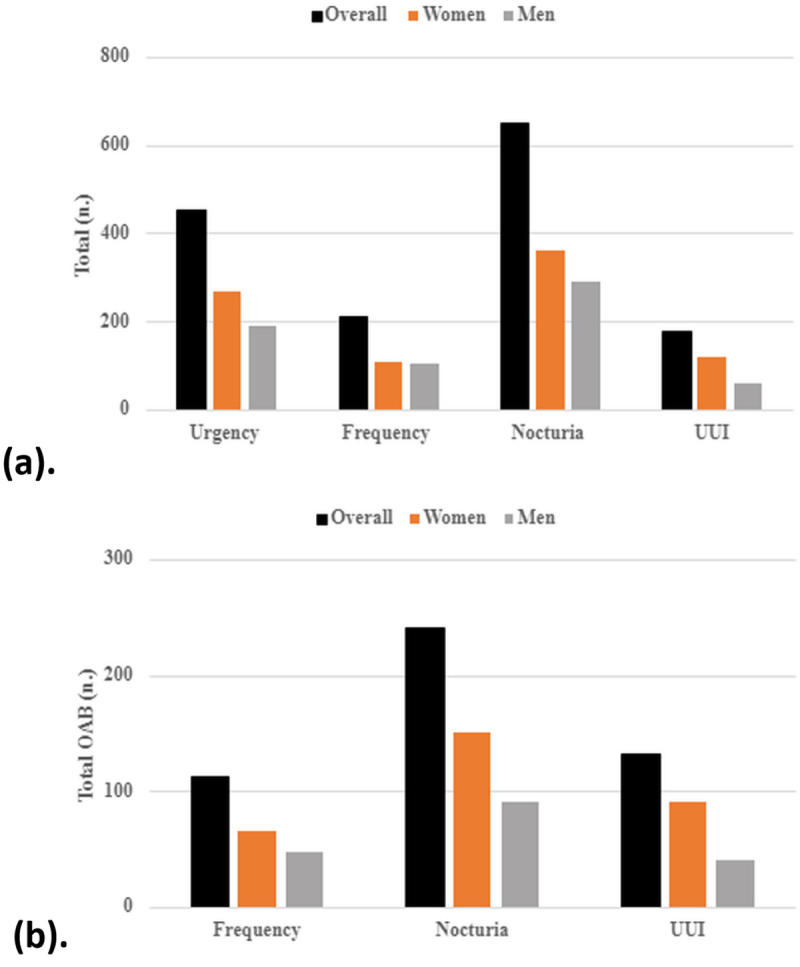
Frequency of OAB symptoms (a)among general population, (b) among OAB group.

Among all respondents, nocturia (defined by the ICS as ≥ 1 voids per night) was the most common symptom (*n* = 650) followed by urgency (*n* = 455), frequency (*n* = 211) and lastly urge UI (*n* = 179) (Figure 1a). Since the diagnosis of OAB in our study entailed the presence of urgency, we aimed to also explore the prevalence of the other spectrum of symptoms. Nocturia remained the most common symptom (*n* = 241) (Figure 1b).

Table 3 illustrates the scores of symptoms bother and HRQL measured by the OAB-q SF and expressed as mean ± (SD). Symptom bother significantly increased from those without OAB and across OAB severity groups (*p* < 0.001). For those without OAB, the calculated mean ± (SD) was 8.9 ± 11.4 while the highest symptom bother was observed in severe OAB (mean ± (SD) = 46 ± 24.4) (Table 3). Regarding HRQL, a significant decrease was noted with the increase in OAB severity (*p* < 0.001). For those without OAB, HRQL mean ± (SD) was 94.9 ± (9.1), compared to 66.7 ± (23.2) in those with severe OAB.

**Table 3.** The Impact of OAB severity on degree of symptom bother and HRQL.

	No OAB	OAB, *n* = 258%)			
		Mild, *n* = 94 (36.4)	Moderate, *n* = 32 (12.4)	Severe, *n* = 132 (51.16)	P value
Symptom bother (mean + SD)	8.9 ± 11.4	21.1 ± 11.9	34.3 ± 16.4	46 ± 24.4	<0.001*
HRQL (mean + SD)	94.9 ± 9.1	83.7 ± 15.1	69.7 ± 22.5	66.7 ± 23.2	<0.001*

*OAB: overactive bladder; HRQL: health-related quality of life*.

*Statistically significant P value < 0.05.

In light of the above mentioned, multivariable linear regression was performed to explore the correlation of each symptom with the degree of bother and HRQL while accounting for the role of age and gender, as illustrated in Table 4. All OAB symptoms showed significant positive correlation with increased symptom bother (*p* < 0.001) in addition to significant inverse correlation with HRQL (*p* < 0.001). For increased symptom bother, urge UI had the highest correlation (β = 6.1, 95% CI: 5.1–7.1, *p* < 0.001), while decrease in HRQL had the highest correlation with daytime frequency (β = −6.8, 95% CI: −8.5 - −5.15, *p* < 0.001).

**Table 4.** Linear regression analysis of OAB symptoms contribution to symptom bother and HRQL as measured by the OABq.

Multivariable linear regression for HRQL (Adjusted R^2^ = 0.56)			Multivariable linear regression for symptom bothers (Adjusted R^2^ = 0.56)	
	β co. efficient (95% CI)	P	β co. efficient (95% CI)	P
Frequency	−6.8 (−8.5 - −5.15)	<0.001*	5.9 (4.4–7.7)	<0.001*
Nocturia	−3.6 (−4.5 - −2.7)	<0.001*	4.4 (3.5–5.4)	<0.001*
Urgency	−4.02 (−4.7 - −3.2)	<0.001*	4.9 (4.1–5.7)	<0.001*
Urge Urinary Incontinence	−4.09 (−5 - −3.1)	<0.001*	6.1 (5.1–7.1)	<0.001*
Age	−0.05 (−0.09 - −0.004)	0.03*	0.005 (−0.04–0.05)	0.8
Gender	0.57 (−0.89–2.04)	0.4	−1.4 (−2.9–0.06)	0.06

*HRQL: health-related quality of life*.

*Statistically significant P value < 0.05.

### Healthcare and treatment seeking behavior

In those with OAB, 18% sought medical advice regarding their urinary condition of which 40% received treatment and 42.5% were diagnosed with OAB. All those who had received treatment reported the use of pharmacotherapy. Among those, 52.6% were currently treated and 47.4% had discontinued their treatment.

## Discussion

To the best of our knowledge, this is the first study conducted in Jordan that explores OAB prevalence in both genders while incorporating broad age groups with assessment of HRQL. Clinical characteristics of those affected were evaluated and the following were significantly associated with increased risk of OAB; female gender, increased BMI, presence of comorbidities and history of recurrent UTIs. In patients with OAB, nocturia was the most common symptom in both genders. Symptom bother significantly increased with the increase in OAB severity and HRQL significantly decreased with the increase in OAB severity All OAB symptoms significantly correlated with increased symptom bother and decreased HRQL, but frequency and urge UI had the highest correlation.

In this study, the prevalence of OAB was 27.4% (22% in men and 32.4% in women) which was comparable to many studies [21–23]. In China, Taiwan and South Korea [4], the prevalence was 20.8%. Among men and women residing in Poland [21], the prevalence was 26.8% and 39.5%, respectively. In the United States, the prevalence was 43% in women and 27% in men [23]. Other studies showed lower prevalence of OAB, Milsom et al. [10] conducted telephone interviews in France, Germany, Italy, Spain and Sweden, and the reported prevalence was 16.6% (range, 12% − 22%). In Korea, the overall prevalence was 12.2% (10% in men and 14.3% in women) [24]. In Germany, Canada, Sweden, Italy and the UK, Irwin et al. [25] reported that the prevalence of OAB was 11.8% (10.8% in men and 12.8% in women).

We also observed higher prevalence in those ≥40 years with overall prevalence of 36.4% (30.8% in men and 41.3% in women).

Epidemiological studies can use different definitions of OAB and although many studies utilized the 2002 ICS definition [1], criteria to establish the diagnosis, targeted populations (in terms of region, gender, and age), type of survey administered, and the implemented methodology can be different and explain the prevalence variance. The higher prevalence reported in the current study can be explained by the regional difference. This is supported by Erwin et al. [12] who reported that the highest number of individuals affected by OAB are in Asia. Evidently, our results similarly compared with other epidemiological studies conducted in Asia [26,27] where OAB was found in 29.9% of men and 51.4% of women. The attributable cause for the higher prevalence in Asia in these studies remained indefinite; however, cultural, social, economic and hygienic differences were suggested [26].

Furthermore, Edwan et al. [16] reported that among women residing in Jordan aged ≥40 years, the prevalence of OAB was 58.8% with approximately half experiencing urge UI. The significant prevalence difference among genders, can be explained by the anatomical differences and obstetrics history in women. In our study, the overall prevalence of UUI among men was 9.1% (41.84% of those with OAB) and 18.2% among women (56.25% of those with OAB). Our results are in line with previous literature regarding urge UI being more common in women [2,22–24]. Nonetheless, these high numbers can be justified by our broad definition of UI; participants who reported an episode of UI once a week or less up to those who reported 5 episodes a day were all included in the category OABwet. We anticipate a lower prevalence if the diagnosis of UUI required more frequent episodes. Studies concerned with male UI have estimated a prevalence of 3.1% to 12.7% highlighting that the prevalence increases with age. Shamliyan et al. And Diokno et al. have also reported that among men experiencing UI, UUI is the most common type affecting 44% of incontinent men [28,29]. In the UREPIK study, Boyle et al. [30] concluded that 89% of men self-reported leakage of urine occurring at least once a week compared to 34% of men who reported 3 or more episodes per week. This adds to our agreement that our high prevalence can be justified by the number of incontinent episodes. Lastly, risk factors for incontinence including age [28,29], diabetes [28] and BMI were also apparent in the OABwet group where 51.2% were aged 60 years or older, 55.38% had a BMI ≥ 30, and 68.2% had diabetes.

In a meta-analysis that evaluated risk factors for OAB; increase in age, and BMI were significantly associated with increased risk of OAB while higher educational level was significantly inversely correlated [31]. The increased risk of OAB with higher BMI, in our study, was in agreement with other reports [32] that explained the association by the increased intraabdominal pressure causing pelvic floor disorders attributable to consequent repetitive pedundal nerve injury. In addition, higher BMI has an increased link with other comorbidities including diabetes which contributes to bladder dysfunction [31–33]. In our study, the prevalence of comorbidities including diabetes, hypertension, anxiety, depression and recurrent UTIs was significantly higher in the OAB group, this was also in line with the current literature [3,4,9]. Our results showed possible association between increased fluid intake (>2500 mL) and risk of OAB. In a systemic review to identify the role of fluid intake modification on OAB symptoms, the results varied and while some studies showed that decreasing fluid intake can significantly reduce OAB symptoms and vice versa, others reported no correlation [34]. Since individuals with OAB are adopting coping behaviors that include fluid restriction [9,11], proper evaluation is required, to avoid disposing them at higher risks of dehydration and UTIs [34].

Across prior reports, OAB has proven to be a bothersome condition. In our study, the degree of bother significantly increased with OAB severity, which perspectively , increased as the number of symptoms or their episodes amplified. Our findings were supported by Irwin et al. [35] who outlined that the highest percentage of individuals reporting bother was of those affected by urgency and five or more of lower urinary tract symptoms (LUTS). Other studies have also demonstrated that bother is significantly correlated with the frequency of symptoms and severity of OAB [4,5,21]. Our results were also in line with those of Milsom et al. [3] and Coyne et al. [6], where the OAB-q SF was utilized in both for the evaluation of bother and HRQL. Similarly, HRQL was better in those without OAB compared to those affected. and bothersome OAB had the most substantial decrease in HRQL scores observed as compared to those with OAB who did not report bother.

The strength of the study includes being carried out in accordance with the ICS 2002 definition, allowing for standardized assessment of OAB prevalence. We also used validated and reliable measures to establish OAB diagnosis, determine the degree of bother and impact on HRQL. The exclusion criteria eliminate many underlying conditions participating in OAB symptoms from the analysis allowing for more accurate prediction of idiopathic OAB. However, this study had a number of limitations as all OAB symptoms and comorbidities were self-reported and therefore can be over or underestimated. In addition, the measures utilized depend on recall from the prior week for the OABSS and prior month for the OABq – SF subjecting the information to recall bias. Moreover, and despite following a stringent exclusion criteria, sampling might not be representative of the general population as data were collected in hospital setting.

## Conclusion

OAB is a prevalent condition in both men and women. OAB was significantly higher in women; however, the prevalence of OAB and urge UI among men should not be overlooked. OAB is a bothersome condition that has a significant adverse impact on HRQL. Our results denotive that this detrimental effect is not exclusive to urge UI and can be attributed to the other elements in the symptom spectrum of OAB. We found the following factors to be associated with OAB: Gender, increasing BMI, presence of chronic diseases, and history of recurrent UTI. Screening for OAB should be considered during routine clinical visits for early detection of symptoms and possible modification of risk factors to improve the HRQL. Implementing validated and reliable measures for the assessment of OAB aids healthcare providers in optimizing their evaluation and treatment.

## Data Availability

Raw data were generated at Jordan university hospital. Derived data supporting the findings of this study are available from the corresponding author MA on request.

## Abbreviations

(OAB): Overactive bladder
(ICS): The international continence Society
HRQL: Health-related quality of life
Short Form (OAB-q SF): Overactive Bladder Questionnaire
(BMI): Body mass index
(UTI): Urinary tract infection
(BPH): Benign prostatic hyperplasia
(OABSS): The Overactive Bladder Symptom Score
(NCDE): The National Center of Diabetes and Endocrinology
[OR]: Odd ratio
[CI]: Confidence interval
(UUI): Urge urinary incontinence
